# Dietary Factors Associated With the Progression of Gastric Intestinal Metaplasia: A Multicenter, Prospective Cohort Study in a Western Population

**DOI:** 10.14309/ctg.0000000000001006

**Published:** 2026-03-02

**Authors:** N.E.A. Kapteijn, F.E. Marijnissen, J.K.F. Pluimers, I.L. Holster, L.G. Capelle, I. Schot, M.P. Anten, E.M. Witteman, F. ter Borg, J.P.W. Burger, M. Doukas, E.J. Kuipers, J. Honing, M.C.W. Spaander

**Affiliations:** 1 Department of Gastroenterology and Hepatology, Erasmus MC University Medical Center, Rotterdam, the Netherlands;; 2 Department of Gastroenterology and Hepatology, Maasstad Hospital, Rotterdam, the Netherlands;; 3 Department of Gastroenterology and Hepatology, Meander Medical Centre, Amersfoort, the Netherlands;; 4 Department of Gastroenterology and Hepatology, IJsselland Hospital, Capelle aan den IJssel, the Netherlands;; 5 Department of Gastroenterology and Hepatology, Sint Franciscus Hospital, Rotterdam, the Netherlands;; 6 Department of Gastroenterology and Hepatology, Canisius-Wilhelmina Hospital, Nijmegen, the Netherlands;; 7 Department of Gastroenterology and Hepatology, Deventer Hospital, Deventer, the Netherlands;; 8 Department of Gastroenterology and Hepatology, Rijnstate Hospital, Arnhem, the Netherlands;; 9 Department of Pathology, Erasmus University Medical Centre, Rotterdam, the Netherlands.

**Keywords:** gastric cancer, intestinal metaplasia, diet, risk factors, epidemiology, cohort

## Abstract

**INTRODUCTION:**

Although several dietary factors such as high salt intake are linked to progression of gastric intestinal metaplasia (GIM) to gastric cancer (GC) in high-risk countries, their effect on GIM progression in Western populations remains less clear. This study investigates the influence of dietary factors on GIM progression in a Western population.

**METHODS:**

The PROgression and REgression of precancerous GAstric Lesions (PRORGAL) study (2009–2024) is a prospective cohort study of GIM patients undergoing surveillance. The operative link on GIM criteria determined GIM stage, with an increase in operative link on GIM stage reflecting disease progression. Data on family history, medication use, and diet were collected through self-reported questionnaire and by medical records. Multivariate logistic regression was used to identify risk factors of disease progression.

**RESULTS:**

A total of 312 GIM patients were included (median age 61 years, 50.3% men, median follow-up 54 months, IQR 36). Progression occurred in 112 patients (35.9%), with 6 patients (1.9%) developing high-grade dysplasia or GC. High dietary salt consumption (OR 1.67; 95% CI 1.05–2.68, *P* = 0.04), meat ≥6 servings per week (OR 1.25; 95% CI 1.07–1.46, *P* = 0.004) smoking (OR 1.76; 95% CI 1.04–2.68), autoimmune gastritis (OR 2.49; 95%CI 1.04–5.83) and having a positive first-degree family member with GC (OR 2.01; 95%CI 1.20–3.52) were significantly associated with GIM progression. Fish consumption, alcohol intake, and previous *Helicobater pylori* infection showed no significant association with GIM progression.

**DISCUSSION:**

Increased consumption of meat and salt is significantly associated with GIM progression, suggesting that dietary risk factors of GIM progression are similar in low-incidence and high-incidence countries.

## Graphical abstract

**Figure:**
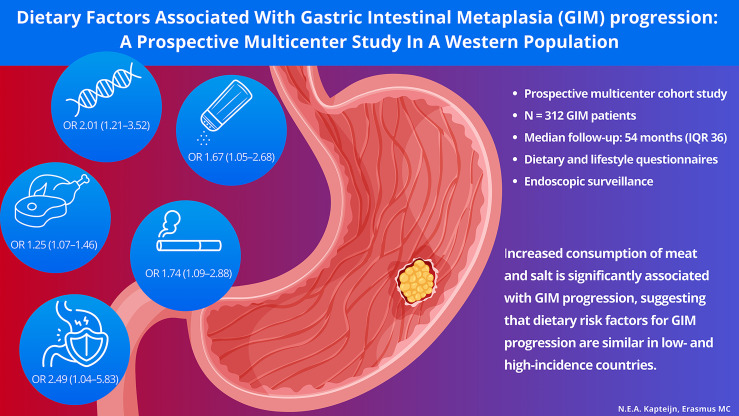

## INTRODUCTION

Gastric cancer (GC) remains one of the most common and deadly cancers worldwide (1). Although a global decline in incidence has been observed due to improved prevention and treatment of *Helicobacter pylori* infection, the absolute number of new cases continues to rise, largely driven by population aging and growth (2). Over 70% of GC cases are reported in developing countries, with the highest incidences observed in Asia and Eastern Europe (3,4). In Western countries, GC incidence is lower compared with high-risk regions; however, mortality rates remain high due to late-stage diagnosis. In the Netherlands, the 5-year survival rate is 21% (5).

One of the key pathways in gastric carcinogenesis is the Correa cascade, a sequence of histopathological changes that progresses from chronic gastritis to atrophic gastritis (AG), gastric intestinal metaplasia (GIM), dysplasia, and ultimately noncardia gastric adenocarcinoma (6,7). These premalignant gastric lesions (PGL) usually arise from (chronic) *H. pylori* infection, which induces gastritis and initiates this sequence of progressive mucosal changes (7). Individuals with an *H. pylori* infection or autoimmune gastritis are at an increased risk of developing GC, in particular when they have premalignant neoplastic lesions (6,8). The updated Management of epithelial precancerous conditions and lesions in the stomach (MAPS II) guideline recommends endoscopic surveillance for high-risk patients, those with AG or GIM involving the gastric antrum and body, older than50 years, a positive family history of GC, persistent *H. pylori* infection, incomplete GIM or AIG (9). In Western populations, the prevalence of GIM ranges from 7% to 15%, depending on age, clinical indication, and risk profile (10,11). Within these low-incidence settings, identifying individuals at increased risk of GC and determining optimal surveillance strategies remains a key clinical challenge. This is due to the lower accuracy of endoscopic detection compared with Asian countries and the low proportion of individuals who ultimately develop GC. This highlights the need to identify additional risk factors associated with the progression of PGLs to GC.

Along with, *H. pylori* infection and age, nonmodifiable risk factors such as sex, ethnicity, and family history are significant contributors to GC risk, especially in high-risk countries (12–14). For example, men generally have a higher risk of developing GC compared with women, and having a first-degree family relative with GC can significantly increase an individual's risk (13,15). However, modifiable risk factors such as lifestyle habits may also play a crucial role in GC development and progression, by influencing shared pathways of chronic mucosal injury, inflammation, and oxidative stress, leading to DNA damage and promotion of a pro-carcinogenic environment (16,17). Although smoking is an established risk factor, (18), and has been previous identified in this cohort (19), dietary factors such as high salt intake and alcohol consumption have also been implicated in gastric carcinogenesis (2,12,13,20). However, most of these studies have been conducted in high-risk Asian countries, where dietary patterns, *H. pylori* prevalence and subtypes, and host factors differ significantly from Western populations. This leaves a critical gap in understanding the impact of these risk factors in low-incidence regions.

The current study uses data from the Progression and Regression of Precancerous Gastric Lesions (PROREGAL) study (21) to investigate the role of dietary factors in the progression of GIM to more advanced neoplastic lesions in the Western population. By identifying modifiable dietary risk factors, this study aimed to contribute to improved risk stratification and targeted prevention strategies for GC in low-risk countries.

## METHODS

### Patient selection

The PROREGAL study is an ongoing multicenter prospective cohort study initiated in August 2009, involving 8 hospitals in the Netherlands. The study protocol received approval from the Erasmus MC Institutional Review Board and Ethics Committee (PROREGAL MEC-2009-090). The study aimed to evaluate the natural history and risk factors associated with the progression and regression of PGLs, including AG, GIM, and dysplasia. In the PROREGAL, individuals aged older than 18 years, diagnosed with PGL are eligible for inclusion. Inclusion criteria for the current study required a diagnosis of extensive GIM (antrum and corpus) or focal GIM (antrum or corpus or incisura) accompanied by at least 1 other risk factor as outlined in the MAPS II guidelines between August 2009 and December 2024 (9). In addition, at least 1 follow-up endoscopy after their initial study endoscopy after index was required. Index endoscopy was defined as first endoscopy with PGL diagnosis and biopsies from both antrum and corpus. Exclusion criteria were: (i) previous upper gastrointestinal surgery, (ii) previous diagnosis of GC or any other malignancy not in remission, (iii) severe comorbid conditions expected to reduce survival by less than 2 years, (iv) portal hypertension, (v) diagnosis of AG or (vii) confirmed presence of a CDH1 mutation. Before inclusion, patients were required to provide written informed consent.

### Data collection

Demographic data including age, sex, and ethnicity were registered for each patient. Medical history, diet, lifestyle factors, medication use, *H. pylori* infection and family history of GC were obtained by structured questionnaires at their initial endoscopy after inclusion, and if possible, at follow-up(s). The questionnaire was self-developed and allowed detailed reporting of dietary and lifestyle factors. The survey consisted of both binary (yes/no) questions and more detailed inquiries regarding specific consumption and lifestyle patterns. Patients were asked about their intake of vegetables and fruits (servings/per day), the consumption of meat (servings/per week), consumption of fish (servings/per week), of prepared meals (servings/per week), the frequency of alcohol consumption (never/current/past and glasses/per week), smoking (never/current/past and cigarettes/per day), and their salt intake (per week), which was self-reported as either low, normal, or high, based on participants' perceived habitual dietary salt consumption. No visual aids or quantitative references (e.g., grams per day) were provided. Smoking and alcohol consumption were included in the model as binary variables (ever vs never), with the ever category encompassing patients who had smoked or consumed alcohol currently or in the past. Portion sizes were not explicitly assessed in the questionnaire. However, expected was that portion sizes were relatively consistent across participants, as meat, fish, vegetables, and fruit are commonly purchased in standardized units and packaging (e.g., grams per portion) in Dutch supermarkets. In addition, patients were queried about whether they were following any specific diets and, if so, which types. Supplement use was also assessed, including the types of vitamins consumed and the corresponding dosages. The questionnaire further inquired about medication use, including nonsteroidal anti-inflammatory drugs (NSAIDs), gastric acid suppressants, and other relevant medications (see Supplementary Table 1, Supplementary Digital Content, http://links.lww.com/CTG/B485).

### Endoscopy procedures

Gastric biopsies were collected systematically at each endoscopy. This included biopsies from any visible lesion and 10 random biopsies from 5 specific regions of the stomach, including 4 quadrant biopsies from the antrum, 2 from the incisura, 2 from the lesser curvature, and 2 from the greater curvature. The time interval of surveillance endoscopies was determined according to the European Society of Gastrointestinal Endoscopy MAPS II guideline, resulting in interval variations between participants. Where available, image-enhanced endoscopy, including narrow band imaging and blue light imaging, was used to optimize mucosal visualization and enable targeted biopsies. Standardized mapping biopsies continued to serve as the basis for operative link on GIM (OLGIM) staging.

### Pathology

The biopsy specimens were fixed in buffered formalin, embedded in paraffin, and subsequently evaluated by gastroenterology expert pathologists from the participating hospitals. The presence and grade of GIM stages were classified using the OLGIM system (22). This system scores GIM as mild, moderate, or severe in both the antrum and corpus, resulting in a stage ranging from 0 to IV, with IV indicating the highest risk of developing GC. Autoimmune gastritis was diagnosed based on serology and histology. The term extensive GIM refers to histological involvement of both antrum and corpus, regardless of grade, and therefore does not necessarily correspond to higher OLGIM stages. All histological assessments were performed by experienced gastrointestinal pathologists using a standardized protocol to minimize interobserver variability.

### Predictor selection

To minimize overfitting in the regression model, we applied an event-per-variable ratio of >10, as commonly recommended in proportional hazards analysis. This means that for every 10 cases of GIM progression, 1 predictor was added to the model, with a maximum of 10 predictors included. Potential predictors were preselected based on existing literature and guidelines, focusing on factors that could be determined before a patient's first upper endoscopy. Previous studies, including our own, have demonstrated a correlation between OLGIM stage and smoking status (21,23), and based on the updated MAPS II guidelines (9), we included a positive first-degree family history of GC, and autoimmune gastritis as potential risk factors of GIM progression in our model. Dietary factors, including meat, fish, salt, fruit, vegetable intake, alcohol consumption, and body mass index, have been investigated in the context of GC, but their potential influence on GIM progression warrants further study (8,24–26). Ethnicity was reported descriptively in baseline characteristics but not included as a predictor due to the predominantly White study population.

### Statistical analyses

Baseline characteristics were presented as mean with SD or median with IQR if not normally distributed. Categorical variables were reported as percentages. The baseline OLGIM stage was compared with follow-up stages to assess GIM progression, using multiple follow-up stages whenever possible or a single follow-up stage if only 1 was available. GIM progression was defined as an increase in OLGIM stage or neoplastic progression on 1 or more endoscopies, depending on the number available. OLGIM progression was defined dichotomously. Neoplastic progression was defined as histologically confirmed development of high-grade dysplasia or gastric adenocarcinoma during follow-up, with the annual neoplastic progression rate calculated as the number of such events divided by the total person-years of follow-up, expressed per 1,000 person-years. We did not expect patients to achieve remission from GIM. Although regression of PGL has been reported in previous studies, this may be due to sampling variability rather than true biological regression. Univariate and multivariable logistic regression analyses were performed to estimate the odds ratio (OR) with 95% 95% CIs for risk factors associated with GIM progression. Missing values were addressed using multiple imputation by chained equations, generating 10 imputed Data sets. Analyses were conducted across imputations using pooled estimates. Multicollinearity was assessed and was not present among the variables included in the final multivariable model. Variables with a univariate *P*-value <0.1 were included in the multivariable analysis. To assess the robustness of the observed associations, univariate relative risk (RR) analyses were performed. The direction and magnitude of the associations were consistent with those observed in the primary logistic regression analyses, thereby supporting the robustness of the conclusions. Statistical significance was defined as *P* < 0.05. Analyses were performed using IBM SPSS v.28.

## RESULTS

### Participant demographics

**Figure 1.**
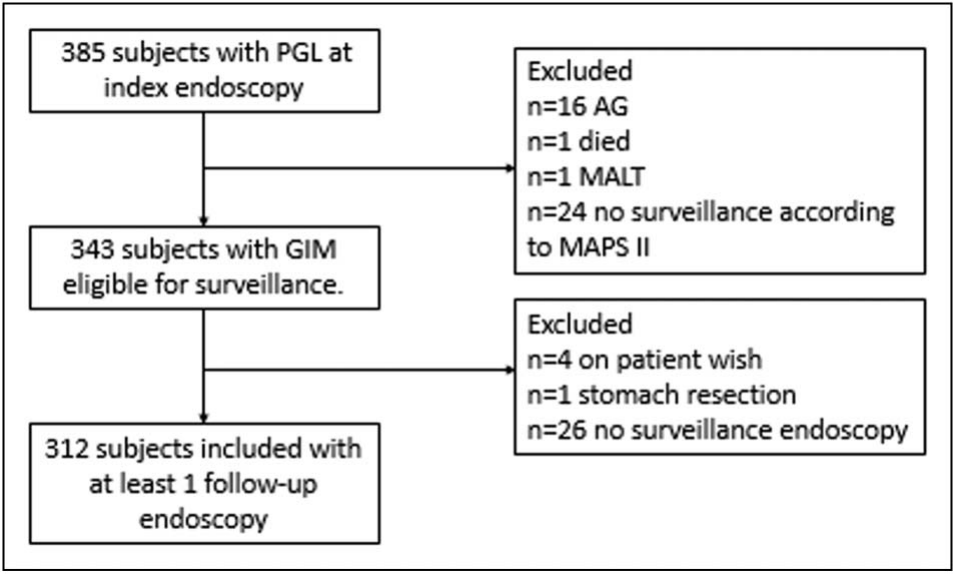
Flowchart of included patients. AG, atrophic gastritis; FU, follow-up; GIM, gastric intestinal metaplasia; IC, informed consent; MALT, mucosa associated lymphoid tissue, MAPS, management of epithelial precancerous conditions and lesions in the stomach; PGL, premalignant gastric lesion.

A total of 385 participants had PGL on index endoscopy and were enrolled in the PROREGAL study. Based on the current study criteria, 40 were excluded because they were diagnosed with AG only, or did not meet the MAPS II guideline criteria for endoscopic surveillance (9). Furthermore, 7 patients were excluded because of mucosa associated lymphoid tissue lymphoma (n = 1), death (n = 1), stomach resection (n = 1), or at patient's request (n = 4) and 26 had not yet undergone their scheduled surveillance endoscopy according to the MAPS II guideline intervals. As a result, 312 patients were included in this study (Figure 1).

**Figure 2.**
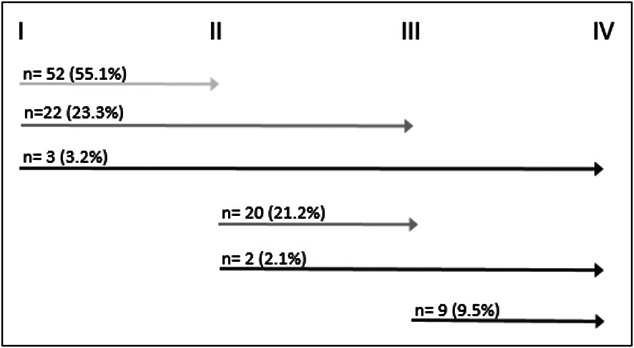
Proportion of subjects with progression per operative link on intestinal metaplasia stage.

The median age of the patients was 61 years (IQR 12), and 50.3% were men (Table 1). Positive family history of GC was documented in 95 participants (31.5%). Fifty-two subjects (17.2%) had a first-degree relative with GC, 30 subjects (9.9%) had a second-degree relative with GC, and in 13 (4.3%) subjects the degree was not categorized. At first inclusion endoscopy, 91,0% (n = 284) was diagnosed with extensive GIM, meaning involvement of the gastric corpus and antrum. The distribution of OLGIM stages was as follows: stage I (37.2%), stage II (39.7%), stage III (18.9%), and stage IV (4.2%). The median follow-up duration was 4.5 years (54 months, IQR 36), during which patients underwent a median of 2 (IQR 1) endoscopies. During this period, most of the cohort (64.1%) exhibited stable disease. Progression was observed in 35.9% (n = 112) of the patients. Among them, 106 patients showed GIM progression with 55.1% progressing to stage II, 44.4% to stage III, and 14.8% to stage IV (Figure 2). In addition, 1.9% (n = 6) of the patients with progression developed high-grade dysplasia (n = 2) or GC (n = 4) within a total of 4.5 follow-up years, resulting in an annual neoplastic progression rate of 4.23 per 1.000 person-years (95% CI: 2.02–6.45). The high proportion of extensive GIM at baseline combined with relatively low OLGIM stages reflects the fact that many patients had slight to mild GIM in both locations, which still corresponds to OLGIM stage I or II.

**Table 1.** Baseline characteristics of study participants

**Baseline characteristics**	n (% from available data)	Available data from cohort n (% from total)
Sex (male), n (%)	157 (50.3)	312 (100.0)
Age at baseline (yr), median (IQR)	61 (12.0)	312 (100.0)
Ethnicity (White), n (%)	256 (82.0)	312 (100.0)
Family history of GC, n (%)	95 (31.5)	302 (96.8)
First degree, n (%)	52 (17.2)	
Second degree, n (%)	30 (9.9)	
Missing degree, n (%)	13 (4.3)	
History of *H. pylori* infection, n (%)	173 (65.3)	265 (84.9)
Autoimmune gastritis, n (%)	30 (9.6)	312 (100.0)
Extended GIM, n (%)	184 (58%)	312 (100.0)
OLGIM at baseline, n (%)		312 (100.0)
I	116 (37.2)	
II	124 (39.7)	
III	59 (18.9)	
IV	13 (4.2)	
Progression of OLGIM stage, n (%)	112 (35.9)	312 (100.0)
Progression to HGD, n (%)	2 (0.6)	312 (100.0)
Progression to GC, n (%)	4 (1.3)	312 (100.0)
I	4 (1.3)	
II	1 (0.3)	
III	1 (0.3)	
IV	0 (0.0)	
Smoking (ever), n (%)	147 (47.9)	307 (98.4)
Alcohol use (yes), n (%)	145 (48.0)	302 (96.8)
BMI, n (%)		258 (82.7)
<18.5	4 (1.3)	
>18.6 to <24.9	97 (31.1)	
>25.0 to <29.9	107 (34.3)	
<30.0 to <34.9	38 (12.2)	
>35.0	12 (3.8)	
Salt intake per week, n (%)		254 (81.4)
Low	60 (23.6)	
Moderate	173 (68.1)	
High	21 (8.3)	
Meat servings per week, n (%)		271 (86.9)
0–2	50 (18.5)	
3–5	116 (42.8)	
6≤	105 (38.7)	
Fish intake per week, median (IQR)	1 (1–2)	241 (77.2)
Vegetable and fruit intake per day, median (IQR)	2 (1–3)	271 (86.9)
Prep meal per week, n (%)	49 (23.1)	212 (67.9)
Vitamin supplements, n (%)	174 (72.2)	241 (77.2)
NSAIDs, n (%)	36 (15.3)	236 (75.6)
Gastric acid suppressors, n (%)	122 (36.3)	237 (75.9)

BMI, body mass index; GC, gastric cancer; GIM, gastric intestinal metaplasia; *H. pylori, Helicobacter pylori;* HGD, high-grade dysplasia; NSAID, nonsteroidal anti-inflammatory drug; OLGIM, operative link for gastritis assessment.

### Risk factors of GIM progression

Table 2 summarizes the correlations between dietary factors and the risk of GIM progression. Meat consumption of 6 or more servings per week was significantly associated with an increased risk of progression (OR 1.23; 95% CI 1.07–1.41, *P* = 0.003). High dietary salt intake (OR 1.69; 95% CI 1.03–2.73, *P* = 0.03) was also a significant predictor of progression and both associations remained significant in the multivariate analysis, with meat consumption of 6 or more times per week (OR 1.25; 95% CI 1.07–1.46, *P* = 0.003), and high salt intake (OR 1.67; 95% CI 1.05–2.68, *P* = 0.04) continuing to show a strong association with progression. Fish, vegetable, and fruit consumption demonstrated a protective effect against GIM progression with OR 0.89 (95% CI 0.68–0.1.15, *P* = 0.28) and OR 0.83 (95% CI 0.70–0.99, *P* = 0.04) respectively. However, the protective effect lost significance after multivariate adjustment. Other dietary factors, such as alcohol consumption (OR 1.34; 95% CI 0.83–2.15, *P* = 0.22) and prepared meal consumption (OR 1.07; 95% CI 0.90–1.29, *P* = 0.52), showed no significant association with GIM progression.

**Table 2.** Multivariate logistic regression analysis of risk factors associated with progression of intestinal metaplasia

**Variables**	Univariate analysis OR (95% CI)	*P*-value	Multivariate analysis OR (95% CI)	*P*-value
Sex (male)	1.12 (0.70–1.78)	0.35		
Age at baseline (yr)	0.99 (0.97–1.01)	0.25		
Family history of GC (first degree)	**1.89 (1.16–3.10)**	**0.01**	**2.01 (1.21–3.52)**	**0.009**
History of *H. pylori* infection	1.06 (0.61–1.83)	0.84		
Autoimmune gastritis (yes)	**2.29 (1.04–5.10)**	**0.04**	**2.49 (1.04–5.83)**	**0.04**
BMI	0.99 (0.97–1.01)	0.48		
Smoking (ever)	**1.64 (1.02–2.64)**	**0.04**	**1.74 (1.09–2.88)**	**0.02**
Alcohol consumption (yes)	1.34 (0.83–2.15)	0.22		
Meat consumption (6 ≤ servings per week)	**1.23 (1.07–1.41)**	**0.003**	**1.25 (1.07–1.46)**	**0.004**
Fish consumption (servings per week)	0.89 (0.68–1.15)	0.28		
Salt consumption (high vs moderate/low)	**1.69 (1.03–2.73)**	**0.03**	**1.67 (1.05–2.68)**	**0.04**
Vegetable and fruit consumption (servings per day)	**0.83 (0.70–0.99)**	**0.04**		
Preprepared meal per week (yes)	1.07 (0.90–1.59)	0.52		
Vitamin use (yes)	**0.59 (0.33–1.01)**	**0.08**		
NSAIDS use (yes)	0.67 (0.31–1.45)	0.43		
Gastric acid suppressor use (yes)	0.91 (0.54–1.54)	0.72		

Bold values indicate statistical significance (*P* < 0.05). BMI, body mass index; GC, gastric cancer; *H. pylori, Helicobacter pylori;* NSAID, nonsteroidal anti-inflammatory drug; OR, odds ratio.

Besides dietary habits, family history of GC, autoimmune gastritis, and smoking were identified as significant risk factors of GIM progression. A significant association was observed between having a first-degree relative with GC (OR 1.89; 95% CI 1.16–3.10, *P* = 0.01). This association remained significant after adjustment for other variables (OR 2.01; 95% CI 1.21–3.52, *P* = 0.009). Autoimmune gastritis was also significantly associated with an increased risk of GIM progression (OR 2.29; 95% CI 1.04–5.10, *P* = 0.04), and this association persisted in the multivariate analysis (OR 2.49; 95% CI 1.04–5.83, *P* = 0.04). Similarly, smoking showed a significant association with GIM progression (OR 1.64; 95% CI 1.02–2.64, *P* = 0.04), which remained significant in the multivariate analysis (OR 1.74; 95% CI 1.09–2.88, *P* = 0.02). Several factors, including sex (male: OR 1.12; 95% CI 0.70–1.78, *P* = 0.35), age (OR 0.99; 95% CI 0.97–1.01, *P* = 0.25), history of *H. pylori* infection (OR 1.06; 95% CI 0.61–1.83, *P* = 0.84), body mass index (OR 0.99; 95% CI 0.97–1.01, *P* = 0.48), NSAID use (OR 0.67; 95% CI 0.31–1.45, *P* = 0.43), vitamin use (OR 0.59; 95% CI 0.33–1.01, *P* = 0.08) and gastric acid suppressor use (OR 0.91; 95% CI 0.54–1.54, *P* = 0.72), were not significantly associated with GIM progression.

### RR analysis for GIM progression

Sex and age were not associated with GIM progression (RR 1.01, 95% CI 0.72–1.43, *P* = 0.94; RR 0.99, 95% CI 0.98–1.01, *P* = 0.27, respectively). A positive family history was associated with a significantly increased risk of GIM progression (RR 1.72, 95% CI 1.22–2.38, *P* = 0.002), as was AIG (RR 1.98, 95% CI 1.29–3.40, *P* = 0.02). No significant association was observed for a history of *H. pylori* infection (RR 1.12, 95% CI 0.71–1.97, *P* = 0.78), body mass index (RR per unit increase 0.99, 95% CI 0.92–1.06, *P* = 0.79), alcohol consumption (RR 1.32, 95% CI 0.89–1.55, *P* = 0.54), prepared meal consumption (RR per category increase 1.02, 95% CI 0.88–1.17, *P* = 0.82), NSAID use (RR 0.78, 95% CI 0.31–1.95, *P* = 0.60), vitamin supplementation (RR 0.70, 95% CI 0.31–1.20, *P* = 0.10), or gastric acid suppressant use (RR 1.00, 95% CI 0.55–1.83, *P* = 1.00). Smoking was significantly associated with GIM progression (RR 1.58, 95% CI 1.17–2.24, *P* = 0.01). Dietary factors associated with increased risk included frequent meat consumption (≥6 servings/week; RR 1.18, 95% CI 1.05–1.45, *P* = 0.03) and higher salt intake (RR 1.83, 95% CI 1.07–3.15, *P* = 0.029), whereas fruit and vegetable intake was not significantly associated with progression (RR per category increase 0.92, 95% CI 0.67–1.05, *P* = 0.04).

## DISCUSSION

To our knowledge, this is the first multicenter prospective study to provide valuable insights into the dietary risk factors associated with the progression of GIM in a low-incidence GC population. Despite the common belief that dietary risks, especially from pickled and salty foods, primarily affect Asian populations, our findings indicate that these factors may also significantly affect Western populations.

Our results suggest that 6 or more servings of meat (OR 1.25; 95% CI 1.07–1.46, *P* = 0.004) and high dietary salt intake (OR 1.67; 95% CI 1.05–2.68, *P* = 0.04) are significant risk factors of GIM progression. This aligns with previous research on PGL, and GC progression conducted in high-risk populations (25,27–32). Studies have shown that high levels of meat consumption is consistently associated with an increased risk of GC, with Asian studies reporting an OR of 1.24 (95% CI: 1.00–1.53) for red meat, 1.23 (95% CI: 1.06–1.43) for processed meat and OR: 1.30 (95% CI: 1.09–1.55) for total meat consumption (29–31). Moreover, exposure-response analyses indicated a rising risk of GC with higher consumption of both processed and red meat, with the highest odds ratio observed at a red meat intake of 150 g/d (OR 1.85; 95% CI: 1.56–2.20) (30). Meat can play a key role in a balanced diet by supplying essential nutrients such as proteins, amino acids, vitamins, and other micronutrients (30). However, excessively eating meat, especially red and processed meat, can be harmful because it contains compounds such as heterocyclic amines, polycyclic aromatic hydrocarbons, N-nitroso compounds, and heme iron, which could cause DNA damage, promote oxidative and lead to GC (29–32). High salt intake has also been linked to GC, with a large study showing an OR of 1.71 (95% CI 1.51–1.95) for Europe and 1.48 (95% CI 1.37–1.59) for Asia (28). This ratio also aligns with findings from various studies conducted in the Eastern population (24,27,33). High sodium intake can damage the gastric mucosa, leading to AG, which may increase cell proliferation as part of the repair process and increase the probability of endogenous mutations (34,35). By weakening the mucous barrier, it also enhances the carcinogenic effects of *H. pylori* infection (24,27).

Epidemiological studies have found conflicting results on the association between fish consumption and GC (36–38). In our study, no significant trend was observed. This aligns with findings from a systematic review and meta-analysis, where consuming fish at least once a week was linked to a reduced but not statistically significant risk, OR of 0.79 (95% CI, 0.61–1.03) (12). A key limitation of these studies, including ours, is the lack of distinction between salty and nonsalty fish. Nonsalted, fresh fish is often linked to a reduced risk due to its high content of anti-inflammatory and anti-carcinogenic properties (24,33,36), whereas salted fish can increase the risk of GC because it contains high salt levels and nitrosamines, a carcinogenic compound (33,39). In contrast to Mediterranean and Asian countries, fish is not a major source of dietary protein in the Netherlands, where consumption remains relatively low and is often limited to once or twice per week. The Dutch Nutrition Center has recommended eating fish 1 or 2 times per week, reflecting its more limited role in the diet compared with regions with higher fish consumption. In contrast, meat consumption is more prevalent.

Interestingly in our study, vegetable and fruit consumption were associated with a reduced risk of GIM progression (OR 0.83; 95% CI 0.70–0.99, *P* = 0.04), although this association did not remain significant in the multivariate model. The association is in line with findings from other studies that suggested a protective effect similar to our result for vegetables (12,40–42). One possible explanation for the lack of significance in our multivariate analysis is the variability in food types, preparation methods, and consumption habits, which may have influenced the results. Moreover, power may have been low to detect a significant correlation among our population because of the small numbers of fruit consumption.

The relationship between alcohol consumption and GC has also been extensively examined, particularly in Asian populations. A significant association has been observed, between alcohol intake and the progression of GIM, with a RR of 1.20 (95% CI 1.10–1.31) (17,20,43). In addition, a study conducted in a low-risk population stratified by levels of alcohol consumption, demonstrated a trend where higher daily alcohol intake is associated with an increased risk of GC (OR = 1.20; 95% CI 1.12–1.27) (44). In our study, we did not observe any correlation between alcohol consumption and the progression of GIM. However, it is important to note that our data did not differentiate between types of alcoholic beverages or low and high levels of alcohol consumption, and this may have led to an underestimation of heavy drinking on progression risk within our cohort.

Smoking is another previous identified critical factor in the development of GC (19). A meta-analysis of 232 studies found that current smoking (OR 1.61; 95% CI, 1.49 to 1.75) and former smoking (OR 1.43; 95% CI, 1.29 to 1.59) are associated with an increased risk of GC (12). Our cohort showed a similar association between smoking and GIM progression, solidifying smoking as 1 of the most significant risk factors.

Concerning the use of supplements and medications, our results indicated that vitamin supplements (OR 0.59; 95% CI 0.33–1.01, *P* = 0.08) and NSAIDs (OR 0.67; 95% CI 0.31–1.45, *P* = 0.43) did not statistically significant protect against GIM progression. Previous literature has suggested a protective effect of these factors on GC development (45–47). Unlike these studies, we did not distinguish between different types of supplements and vitamins, which could reveal important correlations with GIM progression (12,45,48). Regarding NSAIDs, the nonsignificance may be attributed to the low frequency of NSAID users in our cohort.

Although age and *H. pylori* infection are consistently associated with GC risk (49–51), their association with progression among patients with established GIM under surveillance seems limited. In a 20-year Colombian high-risk cohort, *H. pylori* eradication was associated with long-term attenuation of histological progression despite previous infection in many patients (52). Population-based cohorts show that older age is associated with higher GC risk after a PGL diagnosis, whereas surveillance data indicate that age has limited independent predictive value for progression once PGLs are established (49,50,53,54). In our cohort we observed no association between disease progression and either age or a history of *H. pylori* infection. Notably, the cohort was relatively older, resulting in limited age variability and potentially reducing the discriminatory value of age within. In addition, *H. pylori* history may inadequately capture biological risk, particularly given the low prevalence of previous infection in our cohort, as persistent infection or eradication status during follow-up is likely more relevant for progression. Together with other determinants, such as autoimmune gastritis or familial susceptibility, this may explain the absence of significant associations.

In our cohort, 35.9% demonstrated progression of GIM over an average follow-up period of 4.5 years, with a neoplastic progression rate of 4.23 per 1.000 person-year (95% CI: 2.02–6.45). GIM progression was evaluated using an increase in OLGIM stage, which accounts for progression within GIM itself (22). As a result, our study reports a higher rate of progression compared with other studies as previous literature tends to define GIM progression as the development of GIM to dysplasia or GC. However, our neoplastic progression rate is consistent with the findings of a recent systematic review, including AG, GIM and dysplasia patients, which found similar progression rates for low- and high-risk countries (55). They reported a neoplastic progression rate of 2.55 per 1,000 person-years (95% CI: 1.38–4.70) in low risk and 4.53 per 1,000 person-years (95% CI: 2.36–8.68) in high-risk populations, with no significant OR difference between the 2 population (55). This highlights the importance of considering other risk factors beyond demographic characteristics.

Although large population-based studies such as European Prospective Investigation into Cancer and Nutrition (EPIC) provide valuable insights into some dietary risk factors for GC, they do not include endoscopic follow-up or standardized surveillance of PGLs (56).

Our study has several limitations. The median follow-up period of 4.5 years might not be sufficient to capture the long-term progression of the disease and its associated risk factors because GC development is usually a slow and ongoing process. Therefore, ongoing research should focus on long-term follow-up to better understand and address risk factors of developing GC. This approach will enable us to identify and target high-risk populations more effectively. In addition, our study design introduces selection bias. Because only patients recommended for endoscopic surveillance according to the recommendations of the MAPS guidelines were included, our study cohort may consist of individuals with a higher baseline risk of neoplastic progression. Relying on self-reported lifestyle and dietary data introduces potential bias because lifestyle changes may occur with progression, affecting our findings' accuracy. Moreover, the subjective nature of salt intake assessment is also a limitation, as perceptions of low and high salt consumption vary between individuals. In addition, our study primarily focused on GIM progression rather than regression as we do not consider this a true possibility, but rather caused by sampling variability. As the PROREGAL study continues and expands, longer-term data with a larger sample size will strengthen our conclusions. Despite these limitations, our study benefits from a multicenter prospective design, comprehensive data collection, and a relatively large sample size of high-risk patients in a low-incidence GC population. Importantly, we recruited histologically confirmed GIM cases and collected dietary information at initial endoscopy.

In conclusion, our prospective multicenter study suggests that dietary factors in particular consuming 6 or more servings of meat per week and high salt intake are risk factors of neoplastic progression in low GC-incidence region, similar to those in high-incidence countries. These findings highlight the importance of dietary adjustments, lifestyle modifications, and targeted interventions in managing and preventing GC globally.

## Lay Summary

This study investigates the impact of dietary factors on gastric intestinal metaplasia (GIM) progression within a Western population, revealing significant associations with meat and salt intake, similar to high-incidence countries.

## CONFLICTS OF INTEREST

**Guarantor of the article:** M.C.W. Spaander, MD, PhD.

**Specific author contributions:** N.E.A.K: conceptualization; data curation; formal analysis; methodology; investigation; resources; visualization; writing – original draft. F.E.M., J.K.F.P.: investigation; resources; writing – review & editing. I.L.H., L.G.C., I.S., M.P.A., E.M.W., F.t.B., J.P.W.B., E.J.K.: resources; writing – review & editing. M.D.: validation; resources; writing – review & editing. J.H., M.C.W.S.: conceptualization; methodology; resources; supervision; writing – review & editing.

**Financial support:** None to report.

**Potential competing interests:** None to report.

**Study Highlights**

**WHAT IS KNOWN**

✓ Gastric intestinal metaplasia is a precursor of gastric cancer.
✓ High salt intake is linked to gastric cancer in high-incidence populations.
✓ Data on diet and GIM progression in Western populations are limited.

**WHAT IS NEW HERE**

✓ In this Western cohort, 35.9% of patients showed GIM progression.
✓ High salt intake was associated with GIM progression.
✓ Frequent meat intake was associated with GIM progression.

## Supplementary Material

## ABBREVIATIONS:

AG: atrophic gastritis
GC: gastric cancer
GIM: gastric intestinal metaplasia
NSAID: nonsteroidal anti-inflammatory drug
OLGIM: operative link on intestinal metaplasia
OR: odds ratio
PGL: premalignant gastric lesion
PROREGAL: PROgression and REgression of precancerous GAstric Lesions
RR: relative risk

## References

[1] Bray F Laversanne M Sung H . Global cancer statistics 2022: GLOBOCAN estimates of incidence and mortality worldwide for 36 cancers in 185 countries. CA: A Cancer J Clinicians 2024;74(3):229–63. 10.3322/caac.21834 38572751

[2] Chen Y-C Malfertheiner P Yu H-T, et al. Global prevalence of *Helicobacter pylori* infection and incidence of gastric cancer between 1980 and 2022. Gastroenterology. 2024;166(4):605–19. 38176660 10.1053/j.gastro.2023.12.022

[3] Shin WS Xie F Chen B . Updated epidemiology of gastric cancer in Asia: Decreased incidence but still a big challenge. Cancers 2023;15(9):2639. 37174105 10.3390/cancers15092639 PMC10177574

[4] Fock KM Ang TL. Epidemiology of *Helicobacter pylori* infection and gastric cancer in Asia. J Gastroenterol Hepatol 2010;25(3):479–86. 20370726 10.1111/j.1440-1746.2009.06188.x

[5] Allemani C Weir HK Carreira H Global surveillance of cancer survival 1995–2009: Analysis of individual data for 25 676 887 patients from 279 population-based registries in 67 countries (CONCORD-2). Lancet (London, England) 2015;385(9972):977–1010. 25467588 10.1016/S0140-6736(14)62038-9 PMC4588097

[6] Huang RJ Choi AY Truong CD . Diagnosis and management of gastric intestinal metaplasia: Current status and future directions. Gut Liver 2019;13(6):596–603. 31394893 10.5009/gnl19181 PMC6860040

[7] Correa P. Human gastric carcinogenesis: A multistep and multifactorial process first American cancer society award lecture on cancer epidemiology and prevention. Cancer Res 1992;52(24):6735–40. 1458460

[8] Castellana C Eusebi LH Dajti E Autoimmune atrophic gastritis: A clinical review. Cancers 2024;16(7):1310. 38610988 10.3390/cancers16071310 PMC11010983

[9] Pimentel-Nunes P Libânio D Marcos-Pinto R, et al. Management of epithelial precancerous conditions and lesions in the stomach (MAPS II): European Society of Gastrointestinal Endoscopy (ESGE), European *Helicobacter* and Microbiota Study Group (EHMSG), European Society of Pathology (ESP), and Sociedade Port. Endoscopy. 2019;51(4):365–88. 30841008 10.1055/a-0859-1883

[10] Sonnenberg AGR Genta RM. Association between *Helicobacter pylori* gastritis and microscopic colitis. Inflamm Bowel Dis. 2016;22(1):182–6. 26383914 10.1097/MIB.0000000000000595

[11] Capelle LGdVA Haringsma J Ter Borg F . T the staging of gastritis with the OLGA system by using intestinal metaplasia as an accurate alternative for atrophic gastritis. Gastrointest Endosc 2010;71(7):1150-8. 20381801 10.1016/j.gie.2009.12.029

[12] Poorolajal J Moradi L Mohammadi Y . Risk factors for stomach cancer: A systematic review and meta-analysis. Epidemiol Health 2020;42:e2020004. 32023777 10.4178/epih.e2020004 PMC7056944

[13] Duc TQ Gotoda T. Identifying high-risk individuals for gastric cancer surveillance from Western and Eastern perspectives: Lessons to learn and possibility to develop an integrated approach for daily practice. World J Gastroenterol 2019;25(27):3546–62. 31367156 10.3748/wjg.v25.i27.3546 PMC6658388

[14] Yusefi AR Bagheri Lankarani K Bastani P . Risk factors for gastric cancer: A systematic review. Asian Pac J Cancer Prev 2018;19(3):591–603. 29579788 10.22034/APJCP.2018.19.3.591 PMC5980829

[15] Feuerstein JD Isaacs KL Schneider Y AGA clinical practice guidelines on the management of moderate to severe ulcerative colitis. Gastroenterology 2020;158(5):1450–61. 31945371 10.1053/j.gastro.2020.01.006 PMC7175923

[16] Nomura AMY Wilkens LR Henderson BE . The association of cigarette smoking with gastric cancer: The multiethnic cohort study. Cancer Causes Control 2012;23(1):51–8. 22037905 10.1007/s10552-011-9854-0 PMC4166441

[17] Joo Kang S Shin CM Sung J . Association between *ALDH2* polymorphism and gastric cancer risk in terms of alcohol consumption: A meta‐analysis. Alcohol Clin Exp Res 2021;45(1):6–14. 33170513 10.1111/acer.14508

[18] Ligato I Dottori L Sbarigia C . Systematic review and meta‐analysis: Risk of gastric cancer in patients with first‐degree relatives with gastric cancer. Aliment Pharmacol Ther 2024;59(5):606–15. 38197125 10.1111/apt.17872

[19] Nieuwenburg SAV Mommersteeg MC Eikenboom EL Factors associated with the progression of gastric intestinal metaplasia: A multicenter, prospective cohort study. Endosc Int Open 2021;9(3):E297–E305. 33655025 10.1055/a-1314-6626 PMC7892268

[20] Jun S Park H Kim U-J Cancer risk based on alcohol consumption levels: A comprehensive systematic review and meta-analysis. Epidemiol Health 2023;45:e2023092. 37905315 10.4178/epih.e2023092 PMC10867516

[21] Den Hollander WJ Holster IL Den Hoed CM Surveillance of premalignant gastric lesions: A multicentre prospective cohort study from low incidence regions. Gut 2019;68(4):585–93. 29875257 10.1136/gutjnl-2017-314498

[22] Capelle LG de Vries AC Haringsma J The staging of gastritis with the OLGA system by using intestinal metaplasia as an accurate alternative for atrophic gastritis. Gastrointest Endosc 2010;71(7):1150–8. 20381801 10.1016/j.gie.2009.12.029

[23] Lee JWJ Zhu F Srivastava S Severity of gastric intestinal metaplasia predicts the risk of gastric cancer: A prospective multicentre cohort study (GCEP). Gut 2022;71(5):854–63. 33975867 10.1136/gutjnl-2021-324057 PMC8995828

[24] Wu B Yang D Yang S . Dietary salt intake and gastric cancer risk: A systematic review and meta-analysis. Front Nutr 2021;8:801228. 34957192 10.3389/fnut.2021.801228 PMC8692376

[25] Xuexian F Xuyan H Peng A . Landscape of dietary factors associated with risk of gastric cancer: A systematic review and dose-response meta-analysis of prospective cohort studies. Eur J Cancer 2015;51(18):2820. 26589974 10.1016/j.ejca.2015.09.010

[26] Bouras E Tsilidis KK Triggi M . Diet and risk of gastric cancer: An umbrella review. Nutrients 2022;14(9):1764. 35565732 10.3390/nu14091764 PMC9105055

[27] Morais S Costa A Albuquerque G Salt intake and gastric cancer: A pooled analysis within the stomach cancer pooling (StoP) project. Cancer Causes Control 2022;33(5):779–91. 35304655 10.1007/s10552-022-01565-y

[28] Wu X Chen L Cheng J . Effect of dietary salt intake on risk of gastric cancer: A systematic review and meta-analysis of case-control studies. Nutrients 2022;14(20):4260. 36296944 10.3390/nu14204260 PMC9609108

[29] Di Y Ding L Gao L . Association of meat consumption with the risk of gastrointestinal cancers: A systematic review and meta-analysis. BMC Cancer 2023;23(1):782. 37612616 10.1186/s12885-023-11218-1 PMC10463360

[30] Ferro A Rosato V Rota M Meat intake and risk of gastric cancer in the stomach cancer pooling (StoP) project. Int J Cancer 2020;147(1):45–55. 31584199 10.1002/ijc.32707 PMC8550819

[31] Kim SR Kim K Lee SA . Effect of red, processed, and white meat consumption on the risk of gastric cancer: An overall and dose–response meta-analysis. Nutrients 2019;11(4):826. 30979076 10.3390/nu11040826 PMC6520977

[32] Zhao Z Yin Z Zhao Q. Red and processed meat consumption and gastric cancer risk: A systematic review and meta-analysis. Oncotarget 2017;8(18):30563–75. 28430644 10.18632/oncotarget.15699 PMC5444765

[33] Yoo JY Cho HJ Moon S Pickled vegetable and salted fish intake and the risk of gastric cancer: Two prospective cohort studies and a meta-analysis. Cancers 2020;12(4):996. 32316595 10.3390/cancers12040996 PMC7225928

[34] Wang XQTP Terry PD Yan H. Review of salt consumption and stomach cancer risk: Epidemiological and biological evidence. World J Gastroenterol 2009;15(18):2204–13. 19437559 10.3748/wjg.15.2204 PMC2682234

[35] Furihata C Ohta H Katsuyama T. Cause and effect between concentration-dependent tissue damage and temporary cell proliferation in rat stomach mucosa by NaCl, a stomach tumor promoter. Carcinogenesis 1996;17(3):401–6. 8631123 10.1093/carcin/17.3.401

[36] Hirabayashi M Wilunda C Murai U Association between fish and shellfish consumption, n-3 polyunsaturated fatty acids, and gastric cancer risk: The Japan public health Center-based prospective study. Eur J Nutr 2024;63(5):1529–44. 38703225 10.1007/s00394-024-03343-9 PMC11329692

[37] Wu S Liang J Zhang L . Fish consumption and the risk of gastric cancer: Systematic review and meta-analysis. BMC Cancer 2011;11(1):26. 21247502 10.1186/1471-2407-11-26 PMC3037921

[38] Yu XFZJ Dong J. Fish consumption and risk of gastrointestinal cancers: A meta-analysis of cohort studies. World J Gastroenterol 2014;20(41):15398-412. 25386090 10.3748/wjg.v20.i41.15398 PMC4223275

[39] Yuesheng QJ-HC Yu W Wang P . Contamination of Chinese salted fish with volatile N-nitrosamines as determined by QuEChERS and gas chromatography–tandem mass spectrometry. Food Chem 2017;232:763-9. 28490138 10.1016/j.foodchem.2017.04.055

[40] Jin G Lv J Yang M Genetic risk, incident gastric cancer, and healthy lifestyle: A meta-analysis of genome-wide association studies and prospective cohort study. Lancet Oncol 2020;21(10):1378–86. 33002439 10.1016/S1470-2045(20)30460-5

[41] Wei N Zhou M Lei S . A meta-analysis and systematic review on subtypes of gastric intestinal metaplasia and neoplasia risk. Cancer Cell Int 2021;21(1):173. 33731114 10.1186/s12935-021-01869-0 PMC7968216

[42] Jarosz M Sekuła W Rychlik E . Impact of diet on long-term decline in gastric cancer incidence in Poland. World J Gastroenterol 2011;17(1):89–97. 21218088 10.3748/wjg.v17.i1.89 PMC3016685

[43] Li Y Eshak ES Shirai K Alcohol consumption and risk of gastric cancer: The Japan collaborative cohort study. J Epidemiol 2021;31(1):30–6. 31902851 10.2188/jea.JE20190304 PMC7738647

[44] Wenting Deng LJ Zhuo H Vasiliou V . Alcohol consumption and risk of stomach cancer: A meta-analysis. Chem Biol Interact 2021;336:109365. 33412155 10.1016/j.cbi.2021.109365

[45] Xi ZJW Zou L. Vitamin D and gastric cancer—A systematic review and meta-analysis. Nutr Hosp 2023;40(5):1080–7. 37334809 10.20960/nh.04410

[46] Sassano M Seyyedsalehi MS Collatuzzo G Dietary intake of vitamin C and gastric cancer: A pooled analysis within the stomach cancer pooling (StoP) project. Gastric Cancer 2024;27(3):461–72. 38436761 10.1007/s10120-024-01476-8 PMC11016516

[47] Wu C-Y Wu M-S Kuo KN, et al. Effective reduction of gastric cancer risk with regular use of nonsteroidal anti-inflammatory drugs in *Helicobacter pylori*–infected patients. J Clin Oncol. 2010;28(18):2952–7. 20479409 10.1200/JCO.2009.26.0695

[48] Larsson SC Mason AM Vithayathil M . Circulating vitamin C and digestive system cancers: Mendelian randomization study. Clin Nutr 2022;41(9):2031–5. 35986965 10.1016/j.clnu.2022.07.040 PMC7613472

[49] De Vries AC Van Grieken NCT Looman CWN . Gastric cancer risk in patients with premalignant gastric lesions: A nationwide cohort study in the Netherlands. Gastroenterology 2008;134(4):945–52. 18395075 10.1053/j.gastro.2008.01.071

[50] Den Hoed CM Kuipers EJ. Gastric cancer: How can we reduce the incidence of this disease? Curr Gastroenterol Rep 2016;18(7):34. 27184043 10.1007/s11894-016-0506-0 PMC4868864

[51] Zhizhilashvili S McHedlishvili I Jankarashvili N . Effect of age at diagnosis on the prognosis of gastric cancer patients: A population-based study in Georgia. Cureus 2024;16(6):e62154. 38993440 10.7759/cureus.62154 PMC11238615

[52] Piazuelo MBBL Mera RM Camargo MC . The Colombian chemoprevention trial: 20-year follow-up of a cohort of patients with gastric precancerous lesions. Gastroenterology 2021;160(4):1106-17.e3. 33220252 10.1053/j.gastro.2020.11.017 PMC7956231

[53] Song H Ekheden IG Zheng Z . Incidence of gastric cancer among patients with gastric precancerous lesions: Observational cohort study in a low risk Western population. BMJ 2015;351:h3867. 26215280 10.1136/bmj.h3867 PMC4516137

[54] Shah SC Wang AY Wallace MB . AGA clinical practice update on screening and surveillance in individuals at increased risk for gastric cancer in the United States: Expert review. Gastroenterology 2025;168(2):405–16.e1. 39718517 10.1053/j.gastro.2024.11.001

[55] Anne I Hahn DTM Huang RJ . Global progression rates of precursor lesions for gastric cancer: A systematic review and meta-analysis. Clin Gastroenterol Hepatol 2024;23(9):1514-24.e13. 39362617 10.1016/j.cgh.2024.09.003 PMC11958785

[56] Cancer IAfRo. European Prospective Investigation into Cancer and Nutrition (EPIC). https://epic.iarc.fr/ Accessed August 10, 2025.

