# Supplementary material for: Dietary Factors Associated With the Progression of Gastric Intestinal Metaplasia: A Multicenter, Prospective Cohort Study in a Western Population
Source: Clin Transl Gastroenterol. 2026 Mar 2;17(5):e01006. doi: 10.14309/ctg.0000000000001006 (PMC13193290; doi:10.14309/ctg.0000000000001006)
Supplement: Supplementary file 1 [file ct9-17-e01006-s001.pdf]

Supplementary table 1. Translated questionnaire on lifestyle, dietary habits, and medical history.

	Question	Response Options
**1**	Date of Birth	(DD/MM/YY)
**2**	Country of Birth	(Open text)
**3**	Ethnicity	(Dutch/Surinamese/Antillean/Turkish/Moroccan/Hindustani/Black/Asian/Other)
**4**	Gender	(Male/Female/Other, prefer not to say)
**5**	Marital Status	(Married/Cohabiting/Single)
**6**	Do you have children?	(Yes, number of children/No)
**7**	Highest level of education	(Primary/Secondary/MBO/HBO/University)
**8**	Occupation	(Open text)
**9**	Do you regularly come into contact with building materials, chemicals, or refined oil products at work?	(Yes/No)
**10**	Height	(cm)
**11**	Weight	(kg)
**12**	Living location	(City/Town/Suburb)
**13**	Type of residence	(Apartment/Porch house/Semi-detached house/Detached house)
**14**	How many people do you live with?	(1-2/3-5/More than 5)

**15**	Do you smoke or have you ever smoked?	(No/Yes, since when? How many cigarettes per day?/Previously smoked, total years)
**16**	Do you drink alcohol?	(No/Yes, glasses per week/Stopped, how many per week before?)
**17**	Do you eat fresh fruits and vegetables daily?	(Yes/No, how many pieces per day?)
**18**	Do you eat ready-made meals?	(No/Yes, how many per week?)
**19**	Do you consume a lot of salt?	(Salt-free/Sometimes a bit of salt/A lot of salt)
**20**	Do you follow a specific diet?	(No/Yes, which one?)
**21**	How often do you eat meat per week?	(Times per week)
**22**	How often do you eat fish per week?	(Times per week)
**23**	Are you under medical treatment (other than an MDL specialist)?	(No/Yes, for which condition?)
**24**	Do you take vitamin supplements or medication?	(No/Yes, which vitamins and/or medication, including name and dosage)
**25**	Have you ever had stomach or esophagus surgery?	(No/Yes, for what?)
**26**	Do you experience any of the following stomach complaints?	(Heartburn/Nausea/Vomiting/Early satiety/Stomach pain - Frequency scale)

**27**	Have you lost weight unintentionally?	(No/Yes, how many kg in how many months?)
**28**	Have you been treated with antibiotics for the Helicobacter bacterium?	(No/Yes, when?)
**29**	Do you have any of the following conditions?	(Barrettes Esophagus/Stomach ulcer/Esophageal varices - Yes/No)
**30**	Do you use NSAIDs?	(No/Yes, which one and how often per week?)
**31**	Do you take acid reducers?	(No/Yes, how often per week?)
**32**	Do you have or have you had an autoimmune disease?	(List of diseases - Select applicable)
**33**	Do autoimmune diseases run in your family?	(Yes/No, which diseases and who in the family?)
**34**	Does your family have a history of the following?	(Stomach cancer/Esophageal cancer/Precursor stages of stomach cancer/Stomach ulcer/Stomach complaints/Helicobacter bacteria - Yes/No, who?)
